# *Sphingomonas* sp. Hbc-6 alters physiological metabolism and recruits beneficial rhizosphere bacteria to improve plant growth and drought tolerance

**DOI:** 10.3389/fpls.2022.1002772

**Published:** 2022-10-28

**Authors:** Fang Wang, Yali Wei, Taozhe Yan, Cuicui Wang, Yinghui Chao, Mingyue Jia, Lizhe An, Hongmei Sheng

**Affiliations:** ^1^ Ministry of Education Key Laboratory of Cell Activities and Stress Adaptations, School of Life Sciences, Lanzhou University, Lanzhou, China; ^2^ Center for Terrestrial Biodiversity of the South China Sea, Hainan University, Haikou, China; ^3^ The College of Forestry, Beijing Forestry University, Beijing, China

**Keywords:** plant growth-promoting bacteria, *Sphingomonas* sp. Hbc-6, drought stress, widely targeted metabolomics, rhizosphere bacterial community

## Abstract

Drought poses a serious threat to plant growth. Plant growth-promoting bacteria (PGPB) have great potential to improve plant nutrition, yield, and drought tolerance. *Sphingomonas* is an important microbiota genus that is extensively distributed in the plant or rhizosphere. However, the knowledge of its plant growth-promoting function in dry regions is extremely limited. In this study, we investigated the effects of PGPB *Sphingomonas* sp. Hbc-6 on maize under normal conditions and drought stress. We found that Hbc-6 increased the biomass of maize under normal conditions and drought stress. For instance, the root fresh weight and shoot dry weight of inoculated maize increased by 39.1% and 34.8% respectively compared with non-inoculated plant, while they increased by 61.3% and 96.3% respectively under drought conditions. Hbc-6 also promoted seed germination, maintained stomatal morphology and increased chlorophyll content so as to enhance photosynthesis of plants. Hbc-6 increased antioxidant enzyme (catalase, superoxide, peroxidase) activities and osmoregulation substances (proline, soluble sugar) and up-regulated the level of beneficial metabolites (resveratrol, etc.). Moreover, Hbc-6 reshaped the maize rhizosphere bacterial community, increased its richness and diversity, and made the rhizosphere bacterial community more complex to resist stress; Hbc-6 could also recruit more potentially rhizosphere beneficial bacteria which might promote plant growth together with Hbc-6 both under normal and drought stress. In short, Hbc-6 increased maize biomass and drought tolerance through the above ways. Our findings lay a foundation for exploring the complex mechanisms of interactions between *Sphingomonas* and plants, and it is important that *Sphingomonas* sp. Hbc-6 can be used as a potential biofertilizer in agricultural production, which will assist finding new solutions for improving the growth and yield of crops in arid areas.

## Introduction

Drought is one of the most common abiotic stresses that seriously threatens the growth and development of plants (Kumar et al., 2021; Mukarram et al., 2021). Generally, water scarcity leads to a series of physiological and metabolic changes in plants, such as limiting photosynthesis and increasing levels of superoxide radicals (*O_2_–* or H_2_O_2_) and malondialdehyde (MDA) which threaten plant health (Ma et al., 2020; Yang et al., 2021) and lead to wilting, dwarfing, and reduction of biomass in plants (Li et al., 2020; Yang et al., 2021). Therefore, drought is a primary factor restricting crop yield (Gupta et al., 2020).

Maize is not only one of the widely cultivated food crops in the world but also an important part of the global grain supply and food security (Hussain et al., 2020; Li Z. et al., 2021). With global warming, drought has caused aggravated damage to agriculture, resulting in a substantial decrease in maize production (Zhang et al., 2019; Ao et al., 2020). Therefore, there is an urgent need for alternative, cheap, natural, and ecofriendly approaches to help maize adapt to drought and reduce crop loss. There is increasing evidence that the utilization of plant growth-promoting bacteria (PGPB) and plant microbiome provides a new perspective in this regard (Liu et al., 2020; Gao et al., 2021; Orozco-Mosqueda et al., 2021).

PGPB play an important role in promoting plant growth and improving plant stress resistance and have increasingly attracted attention, especially with regard to drought stress (Kang et al., 2012; Moreno-Galvan et al., 2020; Abdelaal et al., 2021; Fiodor et al., 2021). PGPB alleviate drought by inducing the accumulation of osmotic regulatory substances in the host plants, reducing leaf conductance and transpiration under drought stress (Malinowski and Belesky, 2000), and promoting root development, thereby increasing the ability of plants to absorb water (Marasco et al., 2013). Certain PGPB protect the cells from oxidative stress by scavenging free radicals and modulating lipid peroxide levels (Mastouri et al., 2010; Lata et al., 2018; Tiepo et al., 2020). Some PGPB regulate primary metabolites (amino acids, etc.) to promote plant growth (Curzi et al., 2008; Hardoim et al., 2008; Aguiar et al., 2016), and improve plant stress resistance by regulating secondary metabolites, such as phenolic compounds, alkaloids, and terpenoids (Planchamp et al., 2015; Xie et al., 2019; Cappellari et al., 2020; Kousar et al., 2020). Moreover, the application of PGPB could affect the rhizosphere microbiome of plants (Zuluaga et al., 2021); however, the PGPB-mediated interaction between plants and their rhizosphere microbiome is still unclear.

The rhizosphere microbiome is crucial for plant productivity due to its essential functions in improving plant nutrient acquisition, disease suppression, and stress tolerance (Santos et al., 2021; Shao et al., 2021). Some plants attract beneficial microorganisms by regulating the synthesis and secretion of specific root exudates, such as triterpenoids (Huang et al., 2019) and benzoxazines (Kudjordjie et al., 2019), to protect the plant under stress conditions, especially pathogen infection (i.e., “cry for help” strategy) (Berendsen et al., 2018; Gao et al., 2021; Liu et al., 2021). Nevertheless, our understanding of the interactions within the complex maize rhizosphere microbiome and how PGPB mediate these relationships under drought still remains unclear.

Most species of *Sphingomonas* possess the ability to degrade a variety of aromatic compounds and industrial pollutants (Leys et al., 2004; Gong et al., 2016; Liu et al., 2017), thus contributing significantly to environmental remediation and industrial production. Recent studies have found that some strains of *Sphingomonas* have the capacity to promote plant growth (Sukweenadhi et al., 2015) and alleviate abiotic stresses (Chen et al., 2014; Khan et al., 2014; Asaf et al., 2018; Luo et al., 2019). However, the knowledge of the interaction between *Sphingomonas* and plants, metabolites, and the rhizosphere microbiome under drought stress is limited.

*Sphingomonas* sp. Hbc-6, isolated from *Nitraria tangutorum* in the desert area of Minqin, Northwest China, is an endophytic bacterium with plant growth-promoting properties and promoting the root development of *Arabidopsis thaliana*. To further explore the mechanism by which Hbc-6 promoted crops growth and improved its drought resistance, we selected maize for subsequent experiments. In this study, we investigated the effects of Hbc-6 on maize phenotype, biomass, physiological metabolism, the rhizosphere bacterial communities and the correlation between metabolome and microbiome under normal conditions and drought stress. We found that *Sphingomonas* sp. Hbc-6 could regulate physiological metabolism, recruit beneficial rhizosphere bacteria, promote plant growth, and ultimately increase plant biomass and drought tolerance.

## Material and methods

### Cultivation of bacteria

*Sphingomonas* sp. Hbc-6 was isolated from the leaves of *N. tangutorum*, a desert plant in Minqin, Gansu, China. The bacterial strains were cultured on R2A agar medium at 28°C. After 60 h of growth, a single colony was picked out and cultured in R2A liquid medium in a rotary shaker (150 rpm) at 28°C for 16 h. Subsequently, bacterial cells were collected *via* centrifugation. Preliminary work found that the inoculation concentration of 1.0~1.5×10^8^ CFU mL^-1^ has the best effect on promoting plant growth, and in order to maintain a consistent inoculation amount each time, the bacterial cells were resuspended in sterile water and adjusted to 1.0~1.5×10^8^ CFU mL^-1^ for using.

### Maize seed germination

The washed maize seeds were sequentially disinfected with 75% ethanol for 3 min and 0.5% sodium hypochlorite solution for 15 min and then washed five times with sterile water. The sterilized seeds were immersed in bacterial solution, and the control was replaced with R2A liquid medium and placed in the incubator (temperature: 26 ± 1°C). After 8 h of cultivation, the seeds were sterilized and washed. The sterilized seeds were then placed in a Petri dish with two layers of filter paper (100 seeds per dish). Sterile water, 5% PEG 6000, 10% PEG 6000, and 15% PEG 6000 solutions were added, in order, which was followed by incubation under a light cycle of 16 h light/8 h darkness at 26 ± 1°C, with light intensity of 5500 lx. The germination rate (GR) was measured for 8 days at an interval of one day. The calculation of GR and germination energy (GE) was done using Chen’s (Chen et al., 2021) method as follows:

GR (%) = number of germinated seeds on day 8/number of all tested seeds × 100
GE (%) = number of germinated seeds on day 3/number of all tested seeds × 100

### Pot experiment

The washed maize seeds were sequentially disinfected with 75% ethanol for 3 min and 0.5% sodium hypochlorite solution for 15 min, washed with sterile water five times, sown on a Petri dish with 2 layers of filter paper, kept wet by adding sterile water, and placed in a light incubator for culture. The coincident germinated seedlings were transplanted into the soil [Pindstrup substrate: roseite (1:2, v/v)]. When the maize seedlings developed three leaves, natural drought and the soil water content was controlled as follows: 60–70% (normal condition, WW), 50–60% (light drought, LD), 40–50% (medium drought, MD), 30–40% (serious drought, HD). After reaching adequate soil water conditions, the roots were irrigated with bacterial solution. Each plant was inoculated with 1 mL of bacterial suspension every day, for seven days, while the control was irrigated with the same volume of sterile water.

### Measurement of plant traits

After seven days of continuous inoculation, the first samples were taken on the first day after inoculation. The leaves from each treatment were observed every five days to determine their physiological indices. The content of chlorophyll was physiologically detected by the method described by Wellburn (1994), and the permeability of the plasma membrane was measured using a conductivity meter (Yang et al., 2011). The content of MDA was determined according to the reactants of thiobarbituric acid (Heath and Packer, 1968). The content of soluble sugar was determined according to the method described by Behrooz et al. (2019). The activities of catalase (CAT), peroxidase (POD), and superoxide dismutase (SOD) were determined according to the methods described by Liang et al. (1982), Amako et al. (1994), and Luo et al. (2019). The 26th day after inoculation, plants were carefully separated from the soil and gently washed with deionized water to remove the attached soil. Then the plant height, root length, fresh weight and dry weight of the aboveground and underground parts were measured. The third leaf from the top of the maize plant was used for observing stomatal morphology according to the method described by Wu et al. (2018).

### Widely targeted metabolism analysis

Whole plants under normal conditions (normal condition with non-inoculation, MC; normal condition with inoculation, WH) and medium drought conditions (medium drought with non-inoculation, DMC; medium drought with inoculation, MH) were collected on the 26th day after inoculation for widely targeted metabolomics evaluation. Maize plants were weighed, divided into 15-mL sterile centrifuge tubes, and stored at -80°C for subsequent experiments. The four groups of samples were crushed using a mixer mill (MM 400, Retsch) with a zirconia bead for 1.5 min at 30 Hz, and 100 mg powder was weighed and extracted overnight at 4°C with 0.6 mL 70% aqueous methanol. Following centrifugation at 10000 ×*g* for 10 min, the extracts were absorbed (CNWBOND Carbon-GCB SPE Cartridge, 250 mg, 3 mL; ANPEL, Shanghai, China, www.anpel.com.cn/cnw) and filtered (SCAA-104, 0.22 μm pore size; ANPEL, Shanghai, China, http://www.anpel.com.cn/) before UPLC-MS/MS analysis. The sample extracts were analyzed using a UPLC-ESI-MS/MS system (UPLC, Shim-pack UFLC SHIMADZU CBM30A system, www.shimadzu.com.cn/; MS, Applied Biosystems). The effluent was alternatively connected to an ESI-triple quadrupole-linear ion trap (QTRAP)-MS. LIT and triple quadrupole (QQQ) scans were acquired on a triple quadrupole-linear ion trap mass spectrometer (Q TRAP), API 4500 Q TRAP UPLC/MS/MS System, equipped with an ESI Turbo Ion-Spray interface, operating in positive and negative ion modes and controlled by Analyst 1.6.3 software (AB Sciex). Instrument tuning and mass calibration were performed with 10 and 100 μmol L^-1^ polypropylene glycol solutions in QQQ and LIT modes, respectively. QQQ scans were acquired as MRM experiments with collision gas (nitrogen) set to 5 psi. DP and CE for individual MRM transitions were performed with further DP and CE optimization. A specific set of MRM transitions was monitored for each period according to the metabolites eluted within this period.

### Bacterial DNA extraction and MiSeq sequencing

The maize rhizosphere soil from normal conditions and medium drought treatment on the 26th day after inoculation were collected and used for high-throughput sequencing. After extracting the DNA of each sample, it was subjected to 1% agarose gel electrophoresis. Specific primers 338F (5′-ACTCCTACGGGAGGCAGCAG-3′) and 806R (5′-GGACTACHVGGGTWTCTAAT-3′) with barcodes were used to amplify 16S rRNA of the bacterial V3-V4 region. The PCR products were detected and quantified by QuantiFluor™-ST Blue Fluorescence Quantification System (Promega), and then, each sample was mixed in the corresponding proportion. The following thermal program was used for amplification: pre-denaturation at 95°C for 3 min, denaturation at 95°C for 30 s, annealing at 50°C for 30 s, extension at 72°C for 45 s (a total of 30 cycles), and finally, extension at 72°C for 10 min. The reaction products were detected using 2% agarose gel electrophoresis. The Miseq library was constructed with TruSeq™ DNA Sample Prep Kit reagents, and the data were optimized using Trimmomatic and FLASH software after sequencing was completed on the Illumina MiSeq sequencing platform (Majorbio, Shanghai, China).

### Statistical analysis

Graphpad Prism 8.0 software (Graphpad software Inc., California, USA) was used for statistical analysis of experimental data (including biomass and physiological data), and two-way analysis of variance (ANOVA) and one-way analysis of variance was performed for significant difference analysis. The differential metabolites were screened based on the combination of fold change and variable importance in project (VIP) value of the orthogonal partial least squares discriminant analysis (OPLS-DA) model (fold change ≥ 2 and ≤ 0.5, VIP ≥ 1). The metabolite spectrum data were analyzed by Analyst 1.6.3 and OPLS-DA. Kyoto Encyclopedia of Genes and Genomes (KEGG) database and Origin 9.0 were employed to analyze the experimental results. Usearch (version 7.1 http://drive5.com/uparse/) was used for the analysis of bioinformatics data of the operational taxonomic units (OTUs) at 97% similarity level. Ribosomal Database Project classifier was used for taxonomic analysis of 97% similar OTU representative sequences, and the Silva database (Release128 http://www.arb-silva.de) was used for bacterial database comparison. Finally, a filtered OTU table was obtained for further analysis. The raw metagenome read data are deposited in the National Center for Biotechnology Information (NCBI) Short Read Archive (BioProject ID: PRJNA816337). Mothur software was used for index analysis, and finally, *R* language was used to analyze and draw the principal coordinates, flora structure, and community heat map.

## Results

### Hbc-6 improved seed germination, maintained leaf stomatal morphology and increased biomass of maize

**Figure 1:**
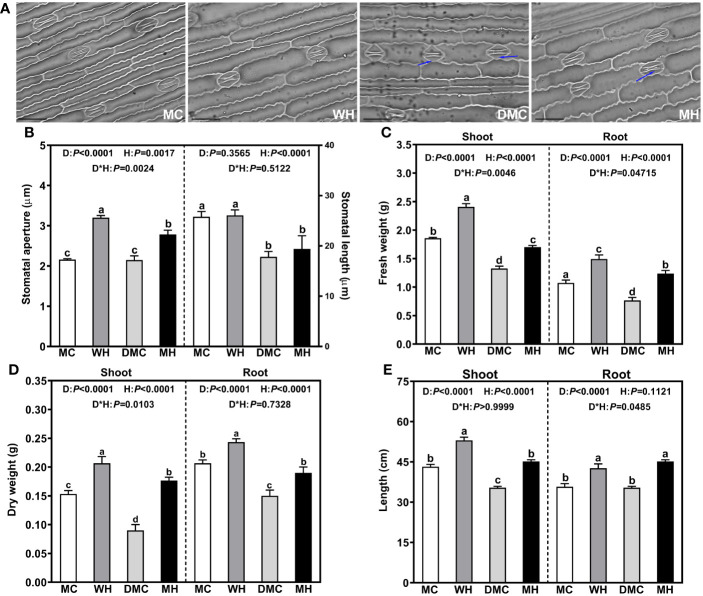
Effects of *Sphingomonas* sp. Hbc-6 inoculation on the stomatal morphology and above- and below-ground biomass of maize under four different treatments, on the 26th day after inoculation with Hbc-6. Representative images of maize stomas **(A)** on the 26th day after inoculation with Hbc-6 under four different treatments. Scale bars represent 100 μm. The blue arrow represent the subsidiary cell of maize. **(B)** Stomatal length and stomatal aperture, **(C)** shoot/root fresh weight, **(D)** shoot/root dry weight, and **(E)** shoot/root length of plants inoculated with Hbc-6 and non-inoculated (control) plants under four different treatments. MC, non-inoculated (control) plants under normal conditions; WH, plants inoculated with Hbc-6 under normal conditions; DMC, non-inoculated (control) plants under medium drought; MH, plants inoculated with Hbc-6 under medium drought. D, drought as factor; H, *Sphingomonas* sp. Hbc-6 as factor; D*H, Interaction between drought and Hbc-6. Data are presented as mean ± standard deviation (SD) of three independent experiments (leaves from three plants). Different letters indicate statistically significant differences (two-way analysis of variance, ANOVA; Tukey test; *p* < 0.05).

Different concentrations of PEG 6000 were applied to simulate drought gradient for exploring the effect of Hbc-6 on seed germination. Plants inoculated with Hbc-6 exhibited significantly higher GR than non-inoculated seeds under 10% PEG 6000 and 15% PEG 6000 treatments, and the GE of inoculated plants was higher than that of non- inoculated plants under no stress and drought stress (**Table S1**). In the pot experiment, our results showed that Hbc-6 alleviated the impact of drought on maize and kept the plants in good health. Here, we observed the stomatal morphology of maize leaves, wherein both ends of the stomatal subsidiary cells began to sharpen after drought treatment (**Figure 1A**), the more severe the drought, the more serious the sharpening of guard cells (**Figure S1A**). Stomatal invagination appeared, stomatal aperture and length significantly decreased, but the stomatal density increased. However, after inoculation with Hbc-6, the stomatal morphology of leaves was restored, the sharpening degree was reduced, and the stomatal aperture was moderately increased (**Figures 1A, B**; **S1**). The fresh weight, dry weight, and plant height of maize significantly decreased under drought stress, but Hbc-6-inoculated maize had significantly higher biomass compared with the non-inoculated plants (**Figures 1C–E**). For example, compared with non inoculation, the root fresh weight, shoot dry weight and plant height of inoculated maize were increased by 61.3%, 96.3% and 27.7% respectively under drought stress (**Figures 1C–E**). In addition, Hbc-6 also significantly increased the biomass of maize under light and severe drought stress (**Figure S1**).

### Hbc-6 improved the drought tolerance of maize by affecting the plant’s physiology and metabolism

**Figure 2:**
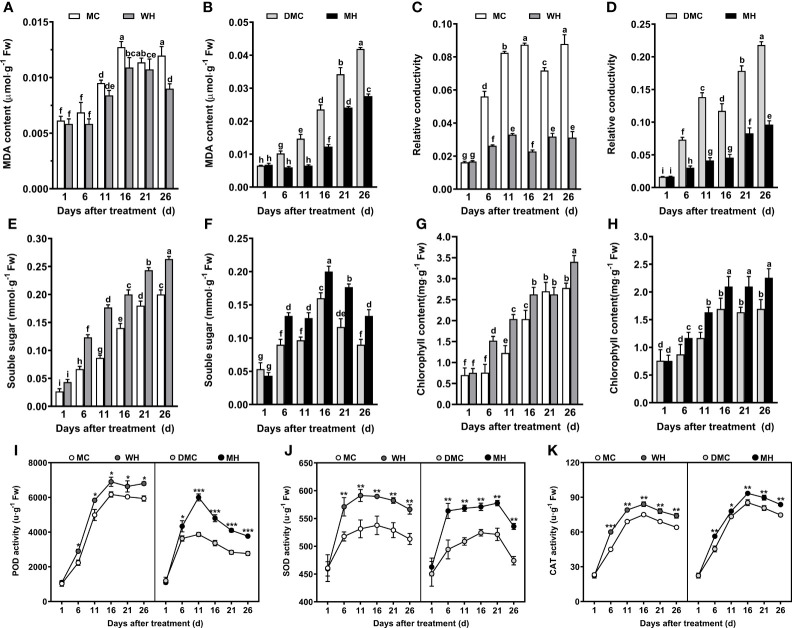
Physiological and antioxidant system responses of maize to *Sphingomonas* sp. Hbc-6 on the 26th day after inoculation under four different treatments. Changes in **(A, B)** MDA content, **(C, D)** relative conductivity, **(E, F)** soluble sugar levels, and **(G, H)** chlorophyll content in maize during the following treatments: MC, WH, DMC, and MH. Time course of **(I)** POD, **(J)** SOD, and **(K)** CAT in response to Hbc-6. MC, non-inoculated (control) plants under normal conditions; WH, plants inoculated with Hbc-6 under normal conditions; DMC, non-inoculated (control) plants under medium drought; MH, plants inoculated with Hbc-6 under medium drought. Data are presented as mean ± standard deviation (SD) of three independent experiments (leaves from three plants). Different letters/asterisks indicate statistically significant differences (one-way analysis of variance, ANOVA; Duncan’s test; *p* < 0.05). *p < 0.05; **p < 0.01; ***p < 0.001.

The results of physiological and biochemical examination of maize leaves showed that MDA content and conductivity increased under normal conditions and drought stress, but compared with the control, inoculation with Hbc-6 effectively reduced the MDA content and conductivity at each time point (**Figures 2A–D**). After inoculation, the content of chlorophyll, soluble sugar, and the activities of three antioxidant enzymes of leaves increased at each time point under normal conditions and drought stress (**Figures 2E–K**). For example, after 11 days of inoculation, soluble sugar content and POD activity increased by 34% and 55.2% respectively compared with that non inoculation under drought stress (**Figures 2F, I** **)**. Similar results were obtained under two other degrees of drought (**Figure S2**).

**Figure 3:**
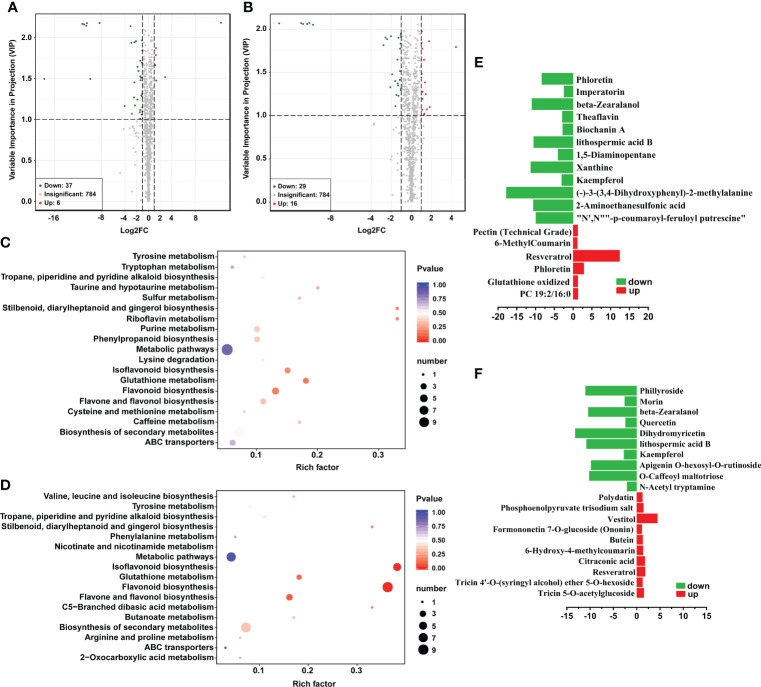
Response of maize metabolites to *Sphingomonas* sp. Hbc-6 on the 26th day after inoculation under four different treatments. **(A)** Volcano plot on differential metabolites in maize of normal group (MC vs. WH) and **(B)** drought group (DMC vs. MH) of medium drought treatment. Kyoto Encyclopedia of Genes and Genomes (KEGG) classification of differential metabolites under normal **(C)** and medium drought **(D)** conditions. The abscissa represents the rich factor corresponding to each path, the ordinate is the pathname, and the color of the point is the *p* value. The red indicates that the enrichment is more significant. The size of the point represents the number of differential metabolites enriched: differential multiples of differentially expressed metabolites on normal **(E)** and medium drought **(F)** conditions. The abscissa represents the log_2_ (fold change), and the ordinate represents the name of metabolite. Red represents upregulated metabolites and green represents downregulated metabolites.

To further analyze the effects of Hbc-6 on maize growth and drought tolerance, we applied widely targeted metabolomics technology to detect the metabolites of maize under normal and medium drought stress conditions. A total of 830 metabolites were detected. The method of combining the VIP value of fold change and the OPLS-DA model (fold change ≥ 2 and fold change ≤ 0.5, VIP ≥ 1) was applied to screen differential metabolites. The results showed that there were 6 upregulated differential metabolites and 37 downregulated substances under normal conditions (MC vs. WH) (**Figure 3A**; **Table S2**), while there were 16 upregulated differential metabolites and 29 downregulated substances in the drought groups (DMC vs. MH) (**Figure 3B**; **Table S3**). KEGG metabolite pathway enrichment analysis revealed that flavonoid biosynthesis, isoflavonoid biosynthesis, and glutathione metabolism were the main metabolic pathways of maize under normal conditions (**Figure 3C**).The enrichment pathway of maize growing in drought were flavonoid biosynthesis, isoflavonoid biosynthesis, flavone and flavonol biosynthesis, and glutathione metabolism (**Figure 3D**). Metabolites such as flavonoids, organic acids and derivatives, amino acids and derivatives, nucleotides and derivatives, vitamins and derivatives, lipids, alkaloids, phenylpropanoids, terpenes, polyphenols, phenolic amines, and quinones were the main differential metabolites both under two conditions (**Tables S2**, **S3**). Compared with non-inoculated plant, Hbc-6 was significantly up-regulated resveratrol and down-regulated beta-zearalanol and other substances under normal conditions (**Figure 3E**; **Table S3**). In addition, resveratrol, putrescine, maleic acid, glutathione, citraconic acid, vestitol and other substances were also upregulated after inoculation compared with the control treatment under drought stress (**Figure 3F**; **Table S3**). Interestingly, resveratrol was upregulated and zeranol was downregulated after inoculation with Hbc-6 both under two conditions (**Figures 3E, F** **)**.

### Hbc-6 altered bacterial community structure of rhizosphere soil and recruited beneficial bacteria

**Figure 4:**
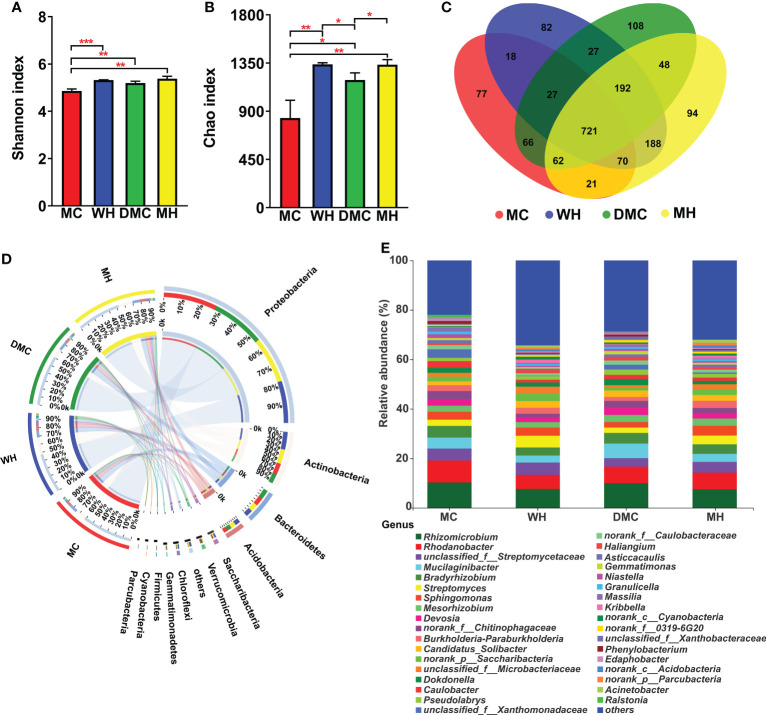
Effects of *Sphingomonas* sp. Hbc-6 on rhizosphere soil bacterial community diversity and composition. **(A)** Shannon index, **(B)** Chao index, and **(C)** Venn diagram of OTU level of rhizosphere bacterial community under normal and medium drought conditions. Relative abundance of rhizosphere soil bacterial community at **(D)** phylum level and **(E)** genus level. Different asterisks indicate significant differences following Student’s t-test (*p < 0.05; **p < 0.01; ***p < 0.001).

**Figure 5:**
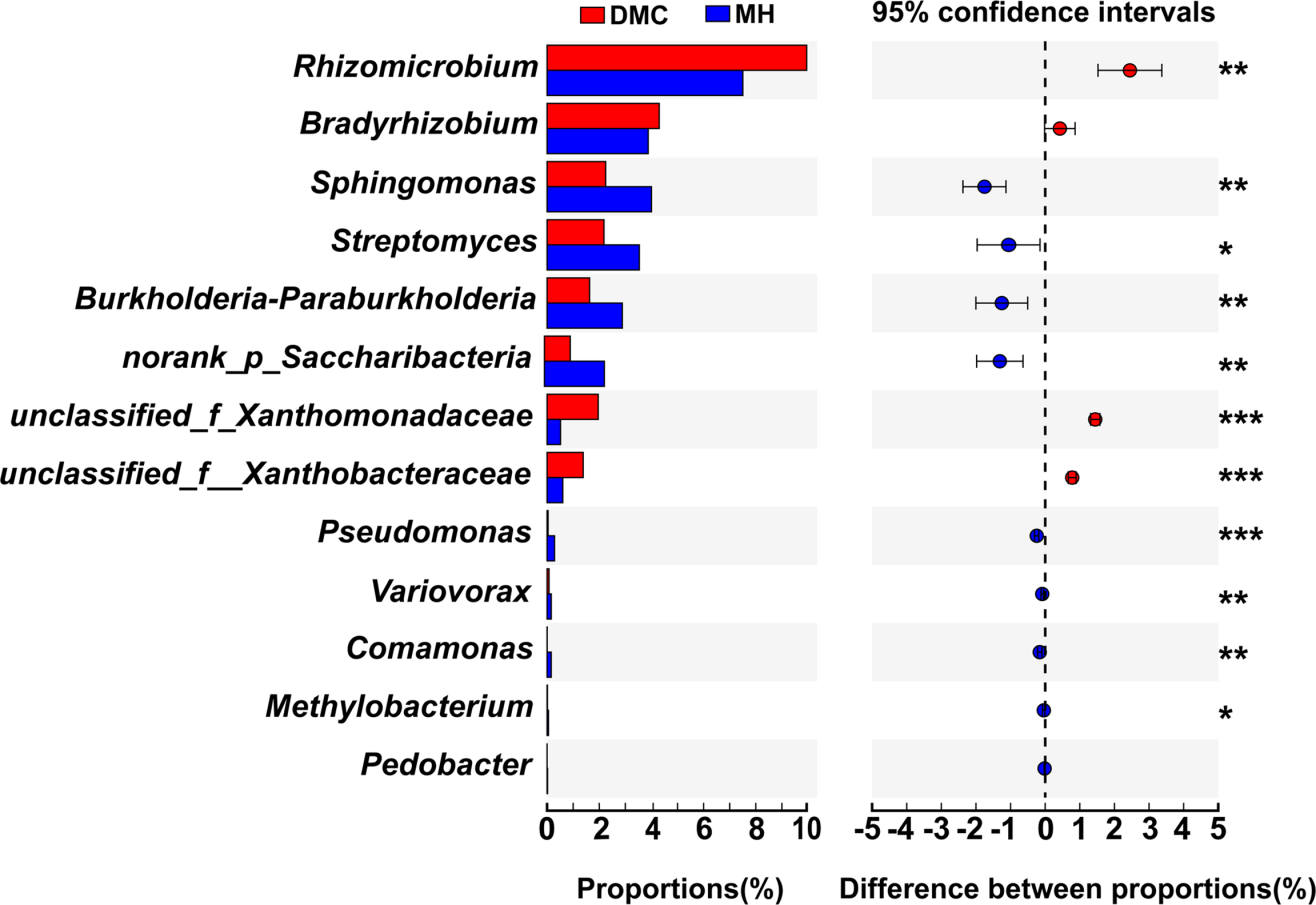
Species difference abundance in rhizosphere soil of inoculated (with Hbc-6) and non-inoculated (control) plants under medium drought at the genus level. The abscissa represents different groups, boxes of different colors represent different groups, and the ordinate represents the average relative abundance of a species in different groups. Different asterisks indicate significant differences following Student’s t-test (*p < 0.05; **p < 0.01; ***p < 0.001).

Bacterial communities in the rhizosphere soil were monitored to investigate the effect of the Hbc-6 on the maize microbiome structure under normal conditions and medium drought stress. The OTUs of rhizosphere soil increased after inoculation with Hbc-6 compared with non-inoculated plants (**Figure 4C**). In particular, the richness and diversity of bacterial communities was significantly lower in non-inoculated rhizosphere soil than in inoculated soil under normal conditions, whereas the richness of bacterial communities significantly increased after inoculation with Hbc-6 under drought stress (**Figures 4A, B**; **S3A, B**). Additionally, inoculation affected the relative abundance of dominant microflora in the rhizosphere soil. The proportions of Actinobacteria and Acidobacteria in the two inoculated groups (WH and MH) were higher than those in the non-inoculated groups (MC and DMC), while the relative abundance of Proteobacteria in the WH and MH groups was lower than that in the non- inoculated groups at phylum level (**Figure 4D**). The abundances of Actinobacteria, Chloroflexi, and Parcubacteria were significantly enhanced after inoculation with Hbc-6 (**Figure 4D**). Analysis of bacterial differential abundance showed that *Streptomyces* and *Cellulomonas* were significantly enriched in inoculated rhizosphere soil under normal conditions (**Figures 4E**, **S3C**), while *Streptomyces*, *Sphingomona*s, *Burkholderia*-*Paraburkholderia*, *Saccharibacteria*, *Pseudomonas*, *Methylobacterium*, *Variovorax*, *Pedobacter*, and *Comamonas* were enriched more under drought stress compared with DMC (**Figures 4E**, **5**). Interestingly, abundance of unclassified *Xanthomonadaceae* members was effectively reduced by inoculation under normal conditions and drought stress.

### Correlations between bacterial communities and metabolites

**Figure 6:**
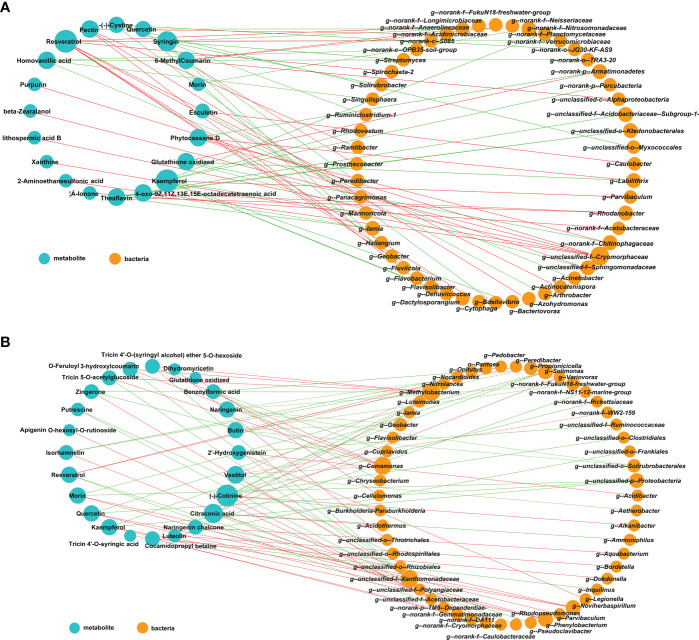
Correlation network of microbiome and metabolome of maize. Correlation network under normal conditions **(A)** and medium drought **(B)**. The blue circle represents metabolites, and the yellow circle represents bacteria at the genus level. The red line represents a significant positive correlation, and the green line represents a significant negative correlation. The thicker the line, the more significant the correlation; the larger the circle, the greater the relative abundance or metabolite expression of microorganisms. We selected differential metabolites and metabolites with correlation > 0.7 and significant correlation test with *p*-value ≤ 0.05.

We further performed Spearman correlation analysis to assess the impact of Hbc-6 on rhizosphere bacterial interactions with the maize plant metabolites. The data showed a significant correlation between different metabolites of maize and some bacteria in the rhizosphere. For example, resveratrol was significantly positively correlated with *Actinocatenispora*, *Cytophaga*, *Dactylosporangium*, and *Geobacter* (*p*< 0.05) in the inoculated group under normal conditions (**Figure 6A**), while resveratrol was significantly positively correlated with beneficial bacteria *Pedobacter* (*p*< 0.05), *Pseudoclavibacter*, and TM6-*Dependentiae* under drought stress (**Figure 6B**). Glutathione was negatively correlated with *Labiltrix*, *Chitinophagaceae*, *Sphingomonadaceae*, and other bacteria (*p*< 0.05) under normal conditions, while glutathione was only significantly positively correlated with *Cellulomonas* (*p*< 0.05) under drought stress (**Figure 6**). Citraconic acid was significantly positively correlated with the beneficial bacterium *Variovorax*; vestitol was significantly positively correlated with *Comamonas* and *Methylobacterium* but significantly negatively correlated with *Xanthomonadaceae* under drought stress (**Figure 6**). These observations were consistent with the amplicon sequencing data and differential metabolite data (**Figures 4**, **5**; **Tables S2**, **S3**). Collectively, these results suggested that Hbc-6 mediated the interactions between rhizosphere microorganisms and maize metabolites.

## Discussion

It was confirmed that inoculation with PGPB regulated plant physiological metabolism and improved plant growth and development (Marasco et al., 2013; Etesami and Maheshwari, 2018). Our study demonstrated that the phenotype, physiology, metabolism, and rhizosphere microbial community of maize significantly changed after inoculation with *Sphingomonas* sp. Hbc-6 and that the biomass and drought tolerance of the plants increased compared with that of non-inoculated plants.

### Physiological and metabolic response mechanism of maize to Hbc-6

Drought is a major threat to crop growth, leading to changes in plant physiological metabolism. For example, drought induces an increase in MDA content and free radical levels in plants which intensifies the damage to the plasma membrane, leads to oxidative stress and endangers the healthy growth of plants (Tsikas, 2017). Additionally, MDA content and cell membrane permeability increase gradually with an increase in the degree and duration of drought stress (**Figures 2**; **S2**). However, the inoculation of Hbc-6 was found to reduce MDA content and cell membrane permeability under drought stress to alleviate the drought-induced damage on plants. The increase of antioxidant enzyme activity and osmotic substance content can promote the growth of plants under drought stress (Scandalios, 1993; Zhang S. H. et al., 2018; Yang et al., 2021). Here, Hbc-6 also promoted plant growth by increasing the activities of SOD, POD, CAT, chlorophyll, soluble sugar, and other beneficial substances under drought stress (**Figures 2**; **S2**).

Metabolites play a crucial role in plant-microbial interaction, plant ecological adaptability, and disease and insect resistance (Hartmann, 2007; Walker et al., 2011). Therefore, we explored the effect of Hbc-6 on maize metabolites under different soil water conditions. The results showed that the quantity of different metabolites changed after inoculation under normal conditions (MC vs. WH) and drought conditions (DMC vs. MH) (**Figures 3E, F**; **Tables S2**, **S3**). This indicated that Hbc-6 affected the metabolites of maize under both soil conditions. For example, resveratrol was upregulated after inoculation compared with the control under the two soil moisture conditions (**Figures 3E, F**). Resveratrol, as a natural plant polyphenol, plays an important antioxidant role with utility in scavenging free radicals, antagonizing pathogens and treating human diseases (Hain et al., 1993; Aziz et al., 2003; Howitz et al., 2003; Kiskova et al., 2020). In addition, putrescine, maleic acid, citraconic acid, and vestitol were upregulated after inoculation with Hbc-6 (**Table S3**) under drought stress. Putrescine is involved in the biological processes of plant growth and abiotic stress response (Evans and Malmberg, 1989) and citraconic acid participates in the TCA cycle (Zhang et al., 2016; Zhang H. L. et al., 2018). Maleic acid improves the metal chelation and antioxidant metabolism of plants, thereby promoting the healthy growth of plants (Al Mahmud et al., 2017). Vestitol, as an antitoxin, effectively antagonizes pathogens and pests (Ueda and Sugimoto, 2010). Hbc-6 inoculation effectively downregulated zeranol (**Figures 3E, F**, **Table S2, S3**) under the two different soil water conditions. Zeranol has strong reproductive toxicity or teratogenicity and destroys the mammalian reproductive system (Sun et al., 2017; Rogowska et al., 2019). Therefore, we propose that Hbc-6 effectively reduces the content of zeranol and provides a safe food source for mammals with a far-reaching significance. Overall, the results of physiology and metabolism suggested that Hbc-6 could improve the adaptability of plants to drought by increasing levels of beneficial substances that promote plant growth and resist stress or by decreasing levels of harmful substances.

### Hbc-6 increased bacterial diversity and recruited more beneficial bacteria in maize rhizosphere soil

Much attention has been drawn to PGPB affecting plant root architecture and physiological metabolism. However, the synergistic effects of PGPB and the rhizosphere microbiome on plants have been rarely studied. Here, we studied the effects of different treatments on the bacterial community structure of the maize rhizosphere *via* high-throughput sequencing of 16S rRNA amplicons. The results showed that the diversity and richness of the bacterial community and OTUs increased after inoculation with Hbc-6 under different soil water conditions (**Figures 4A–C**), and the plants showed higher biomass and healthy growth (**Figure 1**), compared to the growth noted under control treatment. This finding suggested that Hbc-6 could make the bacterial community structure of the rhizosphere more complex and diverse, in order to help plants adapt to adversity and grow well. These observations were consistent with those of other studies (Luo et al., 2019).

After being infected with pathogens, plants recruit microorganisms for protection against disease (Gao et al., 2021; Liu et al., 2021; Yin et al., 2021). Some studies showed that a “call for help” mechanism may also occur during abiotic stress (Santos-Medellín et al., 2017; Liu et al., 2021), which is consistent with our results. Compared with the DMC group, the abundance of *Streptomyces* in the MH group was significantly higher (**Figure 4E**, **Figure 5**). It is known that some *Streptomyces* can produce antibacterial compounds and spores with strong resistance, and the increase in *Streptomyces* was related to the improvement of drought tolerance (Yandigeri et al., 2012; Fitzpatrick et al., 2018), exhibiting potential benefits for host plants (Jones et al., 2017; Worsley et al., 2020). Some recent researches attributed similar benefits to *Sphingomonas*, including plant growth promotion and improvement of resistance to abiotic stress (Asaf et al., 2017; Luo et al., 2019; Wang et al., 2020). After inoculation with Hbc-6 under drought stress, the abundance of *Sphingomonas* significantly increased compared with that in non-inoculated controls. Contrary to previous study results (Qiao et al., 2017), we found that *Sphingomonas* maintained high abundance even after prolonged inoculation (**Figure 4E**, **5**). We propose that Hbc-6 continually plays a key role after inoculation. Additionally, Hbc-6 inoculation significantly increased the abundance of *Burkholderia*, *Paraburkholderia*, *Saccharibacteria*, *Pseudomonas*, *Methylobacterium*, *Variovorax* and *Comamonas*, compared to their abundances in non-inoculated plants under drought stress (**Figures 4E**, **5**). Most of these bacteria have been proved to be beneficial for the healthy growth of plants. For example, *Variovorax* is the core bacterial genus that participates in the development of *Arabidopsis* root system through its auxin degradation operon (Finkel et al., 2020), and *Pseudomonas* and *Methylobacterium* are regarded as more common PGPB (Jorge et al., 2019; Kumar et al., 2019; Liu et al., 2019; Zhang et al., 2019). Moreover, *Saccharibacteria* and *Comamonas* displayed potential for decontamination (Schulze-Makuch et al., 2018) and degradation of chloronitroaromatic pollutants (Liu et al., 2007). These results indicated that Hbc-6 might promote plant growth and improve drought tolerance by recruiting and enriching beneficial bacteria from the rhizosphere.

### Hbc-6 mediated the interaction between plant metabolites and rhizosphere bacteria

Recent studies have focused on the interaction between plant-related microorganisms and plant secondary metabolites (Korenblum et al., 2020; Sun et al., 2021; Xia et al., 2021). An observation confirmed that *Bacillus* in tomato leaf microbiota caused systematic exudation of acylsugar secondary metabolites in tomatoes (Korenblum et al., 2020). Some bacterial communities in the leaf layer of *Cunninghamia lanceolata* were closely related to some types of leaf metabolites, such as alkaloids, aldehydes, vitamins, azoles, and phenols, as reported by Sun et al. (2021). In this study, we found that Hbc-6 mediated the significant correlation between some beneficial bacteria and metabolites under both normal and drought stress conditions (**Figure 6**). For example, an increase in citraconic acid level was significantly positively correlated with *Variovorax*, and an increase in vestitol level was significantly positively correlated with *Comamonas* and *Methylobacterium*, while increases in citraconic acid and vestitol levels were negatively correlated with *Xanthonadaceae*, after inoculation with Hbc-6 under drought stress (**Figure 6**). Citraconic acid was reported to be an intermediate key product of TCA cycle (Zhang et al., 2018), and metabolites of TCA cycle were proved to be able to recruit PGPB in the rhizosphere (Rudrappa et al., 2008; Yuan et al., 2018). Consequently, we speculated that the increased levels of citraconic acid led to the recruitment of, and an increase in abundance of, the beneficial bacterium *Variovorax*, in addition to antagonizing the potentially harmful bacterium *Xanthonadaceae* (Abendroth et al., 2017; Costa et al., 2021), according to the results of 16S amplicon sequencing and differential metabolites (**Figures 3**, **5**). These results revealed the Hbc-6-mediated interaction between plant metabolites and rhizosphere microorganisms. We proposed that Hbc-6, in addition to cooperating with other beneficial bacteria to regulate plant metabolism and improve plant growth ability, directly affected plant metabolite levels to attract beneficial bacteria or antagonize pathogens, thereby promoting plant growth and improving plant drought resistance.

**Figure 7:**
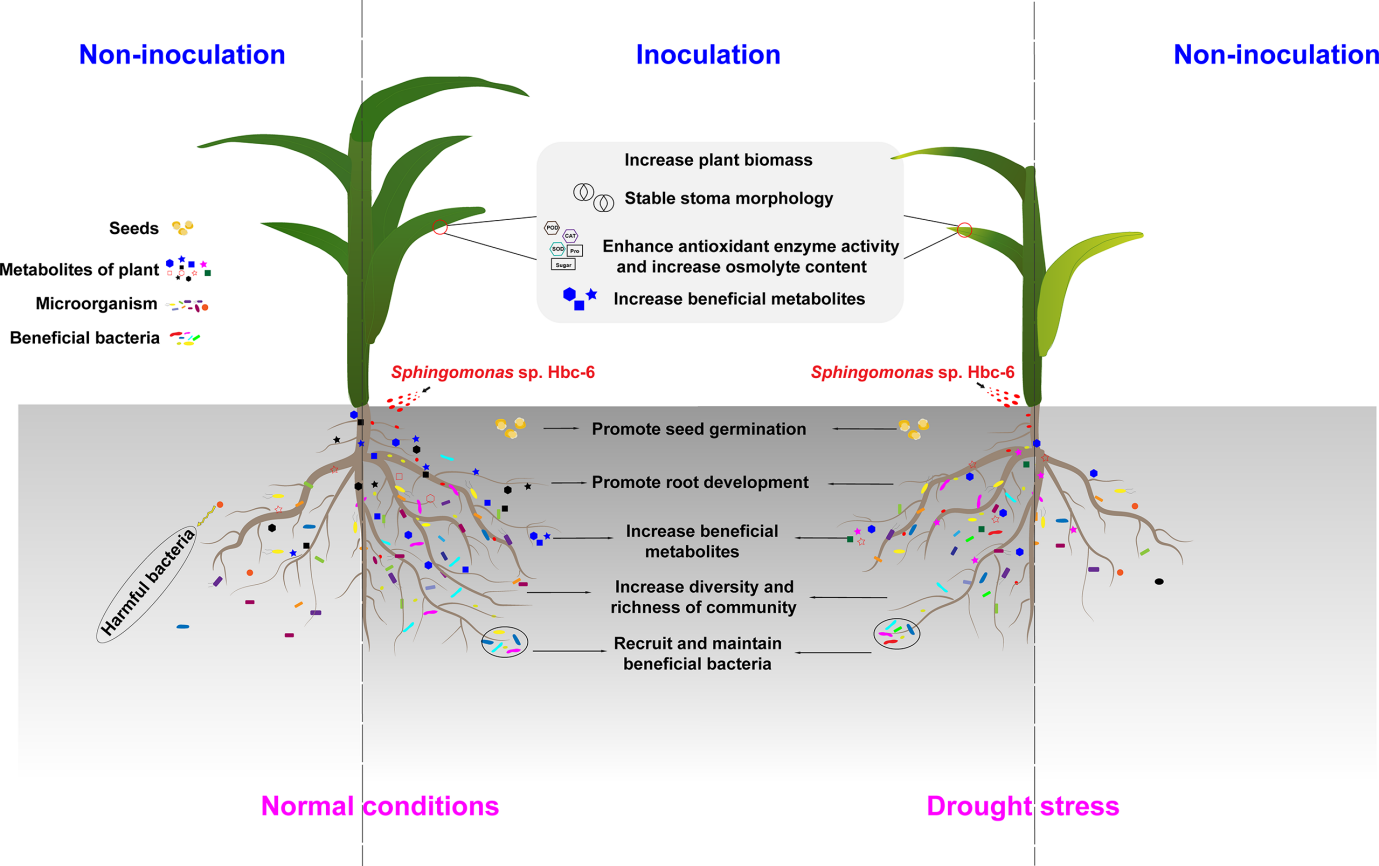
Mechanism of *Sphingomonas* sp. Hbc-6 promoting maize growth and improving drought tolerance.

Based on the above, we found that the mechanism of promoting plant growth and improving plant drought resistance by Hbc-6 was a multi-faceted one (**Figure 7**). Specifically, on the one hand, Hbc-6 promoted seed germination and root development, improved plant photosynthesis (maintenance of stomatal morphology and increase of chlorophyll content), improved antioxidant enzyme activity (SOD, CAT and POD), and increased beneficial osmotic substance content (proline, soluble sugar) under normal conditions and drought stress. In addition, Hbc-6 regulated plant metabolites, upregulated beneficial metabolites (resveratrol, etc.) and down-regulated potentially harmful metabolite (zeranol). These differential metabolites may attract potentially beneficial rhizosphere bacteria, thus promoting plant growth. On the other hand, Hbc-6 reshaped the rhizosphere bacterial community, increased the OTUs and richness and recruited more potentially beneficial bacteria. In a word, Hbc-6 jointly increased maize biomass and improve drought tolerance through the above ways.

## Conclusion

In this study, *Sphingomonas* sp. Hbc-6increased maize biomass, maintained stomatal morphology and regulated physiological metabolism both under normal conditions and drought stress. Additionally, Hbc-6 altered the bacterial community structure of rhizosphere soil, recruited potentially beneficial bacteria and may cooperate with these beneficial bacteria to promote the growth of maize and improve its drought tolerance. However, these potentially beneficial rhizosphere bacteria need further screening and verification of their functions. In a word, our findings provide a theoretical foundation for further understanding of the interaction between *Sphingomonas* and plants under drought stress. Hence, this comprehensive assessment suggests that *Sphingomonas* sp. Hbc-6 is an ecofriendly alternative to chemicals and has high potential to enhance the growth and productivity of maize in arid agroecosystems.

## Data availability statement

The datasets presented in this study can be found in online repositories. The names of the repository/repositories and accession number(s) can be found below: https://www.ncbi.nlm.nih.gov/, PRJNA816337.

## Author contributions

HS, LA, FW and TY conceived and designed the experiments. FW and TY performed the experiments. FW and TY collected the samples and data. FW and TY analyzed the data. FW, HS and YW wrote the manuscript. All authors contributed to revision of the manuscript.

## Funding

This study was financially supported by the National Natural Science Foundation of China (31570488), Major Special Science and Technology Project of Gansu Province (17ZD2WA017).

## Acknowledgments

We also acknowledge Wei Ren (Hainan University) for revising the grammar and language on this manuscript.

## Conflict of interest

The authors declare that the research was conducted in the absence of any commercial or financial relationships that could be construed as a potential conflict of interest.

## Publisher’s note

All claims expressed in this article are solely those of the authors and do not necessarily represent those of their affiliated organizations, or those of the publisher, the editors and the reviewers. Any product that may be evaluated in this article, or claim that may be made by its manufacturer, is not guaranteed or endorsed by the publisher.

## References

[B1] Abdelaal K. AlKahtani M. Attia K. Hafez Y. Kiraly L. Kunstler A. (2021). The role of plant growth-promoting bacteria in alleviating the adverse effects of drought on plants. Biology-Basel 10, 520. doi: 10.3390/Biology10060520 34207963 PMC8230635

[B2] Abendroth U. Adlung N. Otto A. Gruneisen B. Becher D. Bonas U. (2017). Identification of new protein-coding genes with a potential role in the virulence of the plant pathogen *Xanthomonas euvesicatoria* . BMC Genomics 18, 625. doi: 10.1186/S12864-017-4041-7 28814272 PMC5559785

[B3] Aguiar N. O. Medici L. O. Olivares F. L. Dobbss L. B. Torres-Netto A. Silva S. F.. (2016). Metabolic profile and antioxidant responses during drought stress recovery in sugarcane treated with humic acids and endophytic diazotrophic bacteria. Ann. Appl. Biol. 168, 203–213. doi: 10.1111/aab.12256

[B4] Al Mahmud J. Hasanuzzaman M. Nahar K. Rahman A. Hossain M. S. Fujita M.. (2017). Maleic acid assisted improvement of metal chelation and antioxidant metabolism confers chromium tolerance in *Brassica juncea l* . Ecotoxicol. Environ. Safety 144, 216–226. doi: 10.1016/j.ecoenv.2017.06.010 28624590

[B5] Amako K. Chen G. X. Asada K. (1994). Separate assays specific for ascorbate peroxidase and guaiacol peroxidase and for the chloroplastic and cytosolic isozymes of ascorbate peroxidase in plants. Plant Cell Physiol. 35, 497–504. doi: 10.1093/oxfordjournals.pcp.a078621

[B6] Ao S. Russelle M. P. Varga T. Feyereisen G. W. Coulter J. A. (2020). Drought tolerance in maize is influenced by timing of drought stress initiation. Crop Sci. 60, 1591–1606. doi: 10.1002/csc2.20108

[B7] Asaf S. Khan A. L. Khan M. A. Al-Harrasi A. Lee I. J. (2018). Complete genome sequencing and analysis of endophytic *Sphingomonas* sp LK11 and its potential in plant growth. Biotech. 8, 389. doi: 10.1007/S13205-018-1403-Z PMC6111035 30175026

[B8] Asaf S. Khan A. L. Khan M. A. Imran Q. M. Yun B. W. Lee I. J. (2017). Osmoprotective functions conferred to soybean plants *via* inoculation with *Sphingomonas* sp LK11 and exogenous trehalose. Microbiol. Res. 205, 135–145. doi: 10.1016/j.micres.2017.08.009 28942839

[B9] Aziz M. H. Kumar R. Ahmad N. (2003). Cancer chemoprevention by resveratrol: *In vitro* and *in vivo* studies and the underlying mechanisms (Review). Int. J. Oncol. 23, 17–28. doi: 10.3892/ijo.23.1.17 12792772

[B10] Behrooz A. Vandati K. Rejali F. Lotfi M. Sarikhani S. Leslie C. (2019). Arbuscular mycorrhiza and plant growth-promoting bacteria alleviate drought stress in walnut. Hortscience 54, 1087–1092. doi: 10.21273/Hortsci13961-19

[B11] Berendsen R. L. Vismans G. Yu K. Song Y. de Jonge R. Burgman W. P.. (2018). Disease-induced assemblage of a plant-beneficial bacterial consortium. ISME J. 12, 1496–1507. doi: 10.1038/s41396-018-0093-1 29520025 PMC5956071

[B12] Cappellari L. D. Chiappero J. Palermo T. B. Giordano W. Banchio E. (2020). Impact of soil rhizobacteria inoculation and leaf-chewing insect herbivory on *Mentha piperita* leaf secondary metabolites. J. Chem. Ecol. 46, 619–630. doi: 10.1007/s10886-020-01193-3 32577987

[B13] Chen D. W. Hou Q. Z. Jia L. Y. Sun K. (2021). Combined use of two trichoderma strains to promote growth of pakchoi (*Brassica chinensis* l.). Agronomy-Basel 11, 726. doi: 10.3390/Agronomy11040726

[B14] Chen B. Shen J. Zhang X. Pan F. Yang X. Feng Y. (2014). The endophytic bacterium, *Sphingomonas* SaMR12, improves the potential for zinc phytoremediation by its host, *Sedum alfredii* . PloS One 9, e106826. doi: 10.1371/journal.pone.0106826 25198772 PMC4157784

[B15] Costa J. Pothier J. F. Boch J. Stefani E. Jacques M. A. Catara V.. (2021). Integrating science on *Xanthomonadaceae* for sustainable plant disease management in Europe. Mol. Plant Pathol. 22, 1461–1463. doi: 10.1111/mpp.13150 34755430 PMC8578814

[B16] Curzi M. J. Ribaudo C. M. Trinchero G. D. Cura J. A. Pagano E. A. (2008). Changes in the content of organic and amino acids and ethylene production of rice plants in response to the inoculation with *Herbaspirillum seropedicae* . J. Plant Interactions 3, 163–173. doi: 10.1080/17429140802255167

[B17] Etesami H. Maheshwari D. K. (2018). Use of plant growth promoting rhizobacteria (PGPRs) with multiple plant growth promoting traits in stress agriculture: Action mechanisms and future prospects. Ecotoxicol. Environ. Safety 156, 225–246. doi: 10.1016/j.ecoenv.2018.03.013 29554608

[B18] Evans P. T. Malmberg R. L. (1989). Do polyamines have roles in plant development. Annu. Rev. Plant Physiol. Plant Mol. Biol. 40, 235–269. doi: 10.1146/annurev.pp.40.060189.001315

[B19] Finkel O. M. Salas-Gonzalez I. Castrillo G. Conway J. M. Law T. F. Teixeira P. J. P. L.. (2020). A single bacterial genus maintains root growth in a complex microbiome. Nature 587, 103–108. doi: 10.1038/s41586-020-2778-7 32999461 PMC10329457

[B20] Fiodor A. Singh S. Pranaw K. (2021). The contrivance of plant growth promoting microbes to mitigate climate change impact in agriculture. Microorganisms 9:1841, 1–36. doi: 10.3390/microorganisms9091841 PMC8472176 34576736

[B21] Fitzpatrick C. R. Copeland J. Wang P. W. Guttman D. S. Kotanen P. M. Johnson M. T. J. (2018). Assembly and ecological function of the root microbiome across angiosperm plant species. Proc. Natl. Acad. Sci. United States America 115, 1157–1165. doi: 10.1073/pnas.1717617115 PMC5819437 29358405

[B22] Gao M. Xiong C. Gao C. Tsui C. K. M. Wang M. M. Zhou X.. (2021). Disease-induced changes in plant microbiome assembly and functional adaptation. Microbiome 9, 187. doi: 10.1186/s40168-021-01138-2 34526096 PMC8444440

[B23] Gong B. Wu P. Huang Z. Li Y. Dang Z. Ruan B.. (2016). Enhanced degradation of phenol by sphingomonas sp. GY2B with resistance towards suboptimal environment through adsorption on kaolinite. Chemosphere 148, 388–394. doi: 10.1016/j.chemosphere.2016.01.003 26826781

[B24] Gupta A. Rico-Medina A. Cano-Delgado A. I. (2020). The physiology of plant responses to drought. Science 368, 266–269. doi: 10.1126/science.aaz7614 32299946

[B25] Hain R. Reif H. J. Krause E. Langebartels R. Kindl H. Vornam B.. (1993). Disease resistance results from foreign phytoalexin expression in a novel plant. Nature 361, 153–156. doi: 10.1038/361153a0 8421520

[B26] Hardoim P. R. van Overbeek L. S. van Elsas J. D. (2008). Properties of bacterial endophytes and their proposed role in plant growth. Trends Microbiol. 16, 463–471. doi: 10.1016/j.tim.2008.07.008 18789693

[B27] Hartmann T. (2007). From waste products to ecochemicals: fifty years research of plant secondary metabolism. Phytochemistry 68, 2831–2846. doi: 10.1016/j.phytochem.2007.09.017 17980895

[B28] Heath R. L. Packer L. (1968). Photoperoxidation in isolated chloroplasts. i. kinetics and stoichiometry of fatty acid peroxidation. Arch. Biochem. Biophys. 125, 189–198. doi: 10.1016/0003-9861(68)90654-1 5655425

[B29] Howitz K. T. Bitterman K. J. Cohen H. Y. Lamming D. W. Lavu S. Wood J. G.. (2003). Small molecule activators of sirtuins extend *Saccharomyces cerevisiae* lifespan. Nature 425, 191–196. doi: 10.1038/nature01960 12939617

[B30] Huang A. C. C. Jiang T. Liu Y. X. Bai Y. C. Reed J. Qu B. Y.. (2019). A specialized metabolic network selectively modulates *Arabidopsis* root microbiota. Science 364, 546–554. doi: 10.1126/science.aau6389 31073042

[B31] Hussain H. A. Men S. N. Hussain S. Zhang Q. W. Ashraf U. Anjum S. A.. (2020). Maize tolerance against drought and chilling stresses varied with root morphology and antioxidative defense system. Plants-Basel 9, 720. doi: 10.3390/plants9060720 PMC7356637 32517168

[B32] Jones S. E. Ho L. Rees C. A. Hill J. E. Nodwell J. R. Elliot M. A. (2017). *Streptomyces* exploration is triggered by fungal interactions and volatile signals. Elife 6, e21738. doi: 10.7554/eLife.21738 28044982 PMC5207766

[B33] Jorge G. L. Kisiala A. Morrison E. Aoki M. Nogueira A. P. O. Emery R. J. N. (2019). Endosymbiotic *Methylobacterium oryzae* mitigates the impact of limited water availability in lentil (*Lens culinaris medik.*) by increasing plant cytokinin levels. Environ. Exp. Bot. 162, 525–540. doi: 10.1016/j.envexpbot.2019.03.028

[B34] Kang S. M. Khan A. L. Hussain J. Ali L. Kamran M. Waqas M.. (2012). Rhizonin a from burkholderia sp. KCTC11096 and its growth promoting role in lettuce seed germination. Molecules 17, 7980–7988. doi: 10.3390/molecules17077980 22759911 PMC6268351

[B35] Khan A. L. Waqas M. Kang S. M. Al-Harrasi A. Hussain J. Al-Rawahi A.. (2014). Bacterial endophyte *Sphingomonas* sp. LK11 produces gibberellins and IAA and promotes tomato plant growth. J. Microbiol. 52, 689–695. doi: 10.1007/s12275-014-4002-7 24994010

[B36] Kiskova T. Kubatka P. Busselberg D. Kassayova M. (2020). The plant-derived compound resveratrol in brain cancer: A review. Biomolecules 10, 161. doi: 10.3390/Biom10010161 PMC7023272 31963897

[B37] Korenblum E. Dong Y. H. Szymanski J. Panda S. Jozwiak A. Massalha H.. (2020). Rhizosphere microbiome mediates systemic root metabolite exudation by root-to-root signaling. Proc. Natl. Acad. Sci. United States America 117, 3874–3883. doi: 10.1073/pnas.1912130117 PMC7035606 32015118

[B38] Kousar B. Bano A. Khan N. (2020). PGPR modulation of secondary metabolites in tomato infested with *Spodoptera litura* . Agronomy-Basel 10, 778. doi: 10.3390/Agronomy10060778

[B39] Kudjordjie E. N. Sapkota R. Steffensen S. K. Fomsgaard I. S. Nicolaisen M. (2019). Maize synthesized benzoxazinoids affect the host associated microbiome. Microbiome 7, 59. doi: 10.1186/s40168-019-0677-7 30975184 PMC6460791

[B40] Kumar M. Kour D. Yadav A. N. Saxena R. Rai P. K. Jyoti A.. (2019). Biodiversity of methylotrophic microbial communities and their potential role in mitigation of abiotic stresses in plants. Biologia 74, 287–308. doi: 10.2478/s11756-019-00190-6

[B41] Kumar M. Patel M. K. Kumar N. Bajpai A. B. Siddique K. H. M. (2021). Metabolomics and molecular approaches reveal drought stress tolerance in plants. Int. J. Mol. Sci. 22, 9180–9202. doi: 10.3390/Ijms22179108 34502020 PMC8431676

[B42] Lata R. Chowdhury S. Gond S. K. White J. F. (2018). Induction of abiotic stress tolerance in plants by endophytic microbes. Lett. Appl. Microbiol. 66, 268–276. doi: 10.1111/lam.12855 29359344

[B43] Leys N. M. Ryngaert A. Bastiaens L. Verstraete W. Top E. M. Springael D. (2004). Occurrence and phylogenetic diversity of *Sphingomonas* strains in soils contaminated with polycyclic aromatic hydrocarbons. Appl. Environ. Microbiol. 70, 1944–1955. doi: 10.1128/Aem.70.4.1944-1955.2004 15066784 PMC383131

[B44] Liang Z. Yu C. Huang A. H. (1982). Isolation of spinach leaf peroxisomes in 0.25 molar sucrose solution by percoll density gradient centrifugation. Plant Physiol. 70, 1210–1212. doi: 10.1104/Pp.70.4.1210 16662639 PMC1065851

[B45] Li H. La S. K. Zhang X. Gao L. H. Tian Y. Q. (2021). Salt-induced recruitment of specific root-associated bacterial consortium capable of enhancing plant adaptability to salt stress. ISME J. 15, 2865–2882. doi: 10.1038/s41396-021-00974-2 33875820 PMC8443564

[B46] Li Z. Su X. Y. Chen Y. L. Fan X. C. He L. Z. Guo J. M.. (2021). Melatonin improves drought resistance in maize seedlings by enhancing the antioxidant system and regulating abscisic acid metabolism to maintain stomatal opening under PEG-induced drought. J. Plant Biol. 64, 299–312. doi: 10.1007/s12374-021-09297-3

[B47] Liu H. W. Brettell L. E. Qiu Z. G. Singh B. K. (2020). Microbiome-mediated stress resistance in plants. Trends Plant Sci. 25, 733–743. doi: 10.1016/j.tplants.2020.03.014 32345569

[B48] Liu S. Guo C. Dang Z. Liang X. (2017). Comparative proteomics reveal the mechanism of Tween80 enhanced phenanthrene biodegradation by *Sphingomonas* sp. GY2B. Ecotoxicol. Environ. Safety 137, 256–264. doi: 10.1016/j.ecoenv.2016.12.015 27984820

[B49] Liu L. Jiang C. Y. Liu X. Y. Wu J. F. Han J. G. Liu S. J. (2007). Plant-microbe association for rhizoremediation of chloronitroaromatic pollutants with *Comamonas* sp strain CNB-1. Environ. Microbiol. 9, 465–473. doi: 10.1111/j.1462-2920.2006.01163.x 17222144

[B50] Liu R. N. Jiao T. Q. Li J. Feng Y. J. Wang A. Y. Wu S. J.. (2019). Ectopic expression of the *Pseudomonas aeruginosa* KatA gene in cotton improves its drought tolerance and yield under drought stress. Mol. Breeding 39, 117. doi: 10.1007/S11032-019-1027-Y

[B51] Liu H. W. Li J. Y. Carvalhais L. C. Percy C. D. Verma J. P. Schenk P. M.. (2021). Evidence for the plant recruitment of beneficial microbes to suppress soil-borne pathogens. New Phytol. 229, 2873–2885. doi: 10.1111/nph.17057 33131088

[B52] Li W. M. Wang Y. J. Zhang Y. B. Wang R. Y. Guo Z. H. Xie Z. K. (2020). Impacts of drought stress on the morphology, physiology, and sugar content of lanzhou lily (*Lilium davidiivar.unicolor*). Acta Physiol. Plantarum 42, 127. doi: 10.1007/s11738-020-03115-y

[B53] Luo Y. Wang F. Huang Y. L. Zhou M. Gao J. L. Yan T. Z.. (2019). *Sphingomonas* sp. Cra20 increases plant growth rate and alters rhizosphere microbial community structure of *Arabidopsis thaliana* under drought stress. Front. Microbiol. 10. doi: 10.3389/Fmicb.2019.01221 PMC6560172 31231328

[B54] Ma Y. Dias M. C. Freitas H. (2020). Drought and salinity stress responses and microbe-induced tolerance in plants. Front. Plant Sci. 11. doi: 10.3389/Fpls.2020.591911 PMC7691295 33281852

[B55] Malinowski D. P. Belesky D. P. (2000). Adaptations of endophyte-infected cool-season grasses to environmental stresses: Mechanisms of drought and mineral stress tolerance. Crop Sci. 40, 923–940. doi: 10.2135/cropsci2000.404923x

[B56] Marasco R. Rolli E. Vigani G. Borin S. Sorlini C. Ouzari H.. (2013). Are drought-resistance promoting bacteria cross-compatible with different plant models? Plant Signal Behav. 8, e26741. doi: 10.4161/psb.26741 24270625 PMC4091069

[B57] Mastouri F. Bjorkman T. Harman G. E. (2010). Seed treatment with *Trichoderma harzianum* alleviates biotic, abiotic, and physiological stresses in germinating seeds and seedlings. Phytopathology 100, 1213–1221. doi: 10.1094/PHYTO-03-10-0091 20649416

[B58] Moreno-Galvan A. E. Cortes-Patino S. Romero-Perdomo F. Uribe-Velez D. Bashan Y. Bonilla R. R. (2020). Proline accumulation and glutathione reductase activity induced by drought-tolerant rhizobacteria as potential mechanisms to alleviate drought stress in Guinea grass. Appl. Soil Ecol. 147, 103367. doi: 10.1016/J.Apsoil.2019.103367

[B59] Mukarram M. Choudhary S. Kurjak D. Petek A. Khan M. M. A. (2021). Drought: sensing, signalling, effects and tolerance in higher plants. Physiol. Plantarum 172, 1291–1300. doi: 10.1111/ppl.13423 33847385

[B60] Orozco-Mosqueda M. D. Flores A. Rojas-Sanchez B. Urtis-Flores C. A. Morales-Cedeno L. R. Valencia-Marin M. F.. (2021). Plant growth-promoting bacteria as bioinoculants: attributes and challenges for sustainable crop improvement. Agronomy-Basel 11:1167, 1–15. doi: 10.3390/Agronomy11061167

[B61] Planchamp C. Glauser G. Mauch-Mani B. (2015). Root inoculation with *Pseudomonas putida* KT2440 induces transcriptional and metabolic changes and systemic resistance in maize plants. Front. Plant Sci. 5. doi: 10.3389/Fpls.2014.00719 PMC4292437 25628626

[B62] Qiao J. Q. Yu X. A. Liang X. J. Liu Y. F. Borriss R. Liu Y. Z. (2017). Addition of plant-growth-promoting *Bacillus subtilis* PTS-394 on tomato rhizosphere has no durable impact on composition of root microbiome. BMC Microbiol. 17, 1–12. doi: 10.1186/S12866-017-1039-X 28583081 PMC5460418

[B63] Rogowska A. Pomastowski P. Sagandykova G. Buszewski B. (2019). Zearalenone and its metabolites: Effect on human health, metabolism and neutralisation methods. Toxicon 162, 46–56. doi: 10.1016/j.toxicon.2019.03.004 30851274

[B64] Rudrappa T. Czymmek K. J. Pare P. W. Bais H. P. (2008). Root-secreted malic acid recruits beneficial soil bacteria. Plant Physiol. 148, 1547–1556. doi: 10.1104/pp.108.127613 18820082 PMC2577262

[B65] Santos-Medellín C. Edwards J. Liechty Z. Nguyen B. Sundaresan V. (2017). Drought stress results in a compartment-specific restructuring of the rice root-associated microbiomes. Mbio 8, e00764–e00717. doi: 10.1128/mBio.00764-17 28720730 PMC5516253

[B66] Santos S. S. Rask K. A. Vestergard M. Johansen J. L. Prieme A. Froslev T. G.. (2021). Specialized microbiomes facilitate natural rhizosphere microbiome interactions counteracting high salinity stress in plants. Environ. Exp. Bot. 186, 104430. doi: 10.1016/j.envexpbot.2021.104430

[B67] Scandalios J. G. (1993). Oxygen stress and superoxide dismutases. Plant Physiol. 101, 7–12. doi: 10.1104/Pp.101.1.7 12231660 PMC158641

[B68] Schulze-Makuch D. Wagner D. Kounaves S. P. Mangelsdorf K. Devine K. G. de Vera J. P.. (2018). Transitory microbial habitat in the hyperarid atacama desert. Proc. Natl. Acad. Sci. United States America 115, 2670–2675. doi: 10.1073/pnas.1714341115 PMC5856521 29483268

[B69] Shao J. H. Miao Y. Z. Liu K. M. Ren Y. Xu Z. H. Zhang N.. (2021). Rhizosphere microbiome assembly involves seed-borne bacteria in compensatory phosphate solubilization. Soil Biol. Biochem. 159, 108273. doi: 10.1016/j.soilbio.2021.108273

[B70] Sukweenadhi J. Kim Y. J. Kang C. H. Farh Mel A. Nguyen N. L. Hoang V. A.. (2015). *Sphingomonas panaciterrae* sp. nov., a plant growth-promoting bacterium isolated from soil of a ginseng field. Arch. Microbiol. 197, 973–981. doi: 10.1007/s00203-015-1134-z 26163005

[B71] Sun K. Sun H. G. Qiu Z. H. Liu Q. (2021). Comparative analyses of phyllosphere bacterial communities and metabolomes in newly developed needles of *Cunninghamia lanceolata* (Lamb.) hook at four stages of stand growth. Front. Plant Sci. 12. doi: 10.3389/Fpls.2021.717643 PMC8505725 34650578

[B72] Sun X. Tang Q. Du X. L. Xi C. X. Tang B. B. Wang G. M.. (2017). Simultaneous determination of ractopamine, chloramphenicol, and zeranols in animal-originated foods by LC-MS/MS analysis with immunoaffinity clean-up column. Food Analytical Methods 10, 3239–3246. doi: 10.1007/s12161-017-0858-6

[B73] Tiepo A. N. Constantino L. V. Madeira T. B. Goncalves L. S. A. Pimenta J. A. Bianchini E.. (2020). Plant growth-promoting bacteria improve leaf antioxidant metabolism of drought-stressed Neotropical trees. Planta 251, 83. doi: 10.1007/S00425-020-03373-7 32189086

[B74] Tsikas D. (2017). Assessment of lipid peroxidation by measuring malondialdehyde (MDA) and relatives in biological samples: Analytical and biological challenges. Analytical Biochem. 524, 13–30. doi: 10.1016/j.ab.2016.10.021 27789233

[B75] Ueda H. Sugimoto Y. (2010). Vestitol as a chemical barrier against intrusion of parasitic plant *Striga hermonthica* into *Lotus japonicus* roots. Biosci. Biotechnol. Biochem. 74, 1662–1667. doi: 10.1271/bbb.100285 20699571

[B76] Walker V. Bertrand C. Bellvert F. Moenne-Loccoz Y. Bally R. Comte G. (2011). Host plant secondary metabolite profiling shows a complex, strain-dependent response of maize to plant growth-promoting rhizobacteria of the genus *Azospirillum* . New Phytol. 189, 494–506. doi: 10.1111/j.1469-8137.2010.03484.x 20946131

[B77] Wang Q. Ge C. F. Xu S. A. Wu Y. J. Sahito Z. A. Ma L. Y.. (2020). The endophytic bacterium *Sphingomonas* SaMR12 alleviates cd stress in oilseed rape through regulation of the GSH-AsA cycle and antioxidative enzymes. BMC Plant Biol. 20, 63. doi: 10.1186/s12870-020-2273-1 32028891 PMC7006384

[B78] Wellburn A. R. (1994). The spectral determination of chlorophyll-a and chlorophhyll-b, as well as total carotenoids, using various solvents with spectrophotometers of different resolution. J. Plant Physiol. 144, 307–313. doi: 10.1016/S0176-1617(11)81192-2

[B79] Worsley S. F. Newitt J. Rassbach J. Batey S. F. D. Holmes N. A. Murrell J. C.. (2020). *Streptomyces* endophytes promote host health and enhance growth across plant species. Appl. Environ. Microbiol. 86, e01053–e01020. doi: 10.1128/AEM.01053-20 32561579 PMC7414947

[B80] Wu L. M. Li X. Ma L. M. Borriss R. Wu Z. Gao X. W. (2018). Acetoin and 2, 3-butanediol from *Bacillus amyloliquefaciens* induce stomatal closure in *Arabidopsis thaliana* and *Nicotiana benthamiana* . J. Exp. Bot. 69, 5625–5635. doi: 10.1093/jxb/ery326 30295868

[B81] Xia Z. C. He Y. Yu L. Li Z. J. Korpelainen H. Li C. Y. (2021). Revealing interactions between root phenolic metabolomes and rhizosphere bacterial communities in *Populus euphratica* plantations. Biol. Fertility Soils 57, 421–434. doi: 10.1007/s00374-020-01527-z

[B82] Xie Z. C. Chu Y. K. Zhang W. J. Lang D. Y. Zhang X. H. (2019). *Bacillus pumilus* alleviates drought stress and increases metabolite accumulation in *Glycyrrhiza uralensis* fisch. Environ. Exp. Bot. 158, 99–106. doi: 10.1016/j.envexpbot.2018.11.021

[B83] Yandigeri M. S. Meena K. K. Singh D. Malviya N. Singh D. P. Solanki M. K.. (2012). Drought-tolerant endophytic actinobacteria promote growth of wheat (*Triticum aestivum*) under water stress conditions. Plant Growth Regulation 68, 411–420. doi: 10.1007/s10725-012-9730-2

[B84] Yang X. Y. Lu M. Q. Wang Y. F. Wang Y. R. Liu Z. J. Chen S. (2021). Response mechanism of plants to drought stress. Horticulturae 7, 50. doi: 10.3390/horticulturae7030050

[B85] Yang S. Tang X. F. Ma N. N. Wang L. Y. Meng Q. W. (2011). Heterology expression of the sweet pepper CBF3 gene confers elevated tolerance to chilling stress in transgenic tobacco. J. Plant Physiol. 168, 1804–1812. doi: 10.1016/j.jplph.2011.05.017 21724293

[B86] Yin C. T. Vargas J. M. C. Schlatter D. C. Hagerty C. H. Hulbert S. H. Paulitz T. C. (2021). Rhizosphere community selection reveals bacteria associated with reduced root disease. Microbiome 9, 86. doi: 10.1186/S40168-020-00997-5 33836842 PMC8035742

[B87] Yuan J. Zhao J. Wen T. Zhao M. L. Li R. Goossens P.. (2018). Root exudates drive the soil-borne legacy of aboveground pathogen infection. Microbiome 6, 156. doi: 10.1186/S40168-018-0537-X 30208962 PMC6136170

[B88] Zhang H. L. Du W. C. Peralta-Videa J. R. Gardea-Torresdey J. L. White J. C. Keller A.. (2018). Metabolomics reveals how cucumber (*Cucumis sativus*) reprograms metabolites to cope with silver ions and silver nanoparticle-induced oxidative stress. Environ. Sci. Technol. 52, 8016–8026. doi: 10.1021/acs.est.8b02440 29898596

[B89] Zhang S. H. Xu X. F. Sun Y. M. Zhang J. L. Li C. Z. (2018). Influence of drought hardening on the resistance physiology of potato seedlings under drought stress. J. Integr. Agricult. 2, 336–347. doi: 10.1016/S2095-3119(17)61758-1

[B90] Zhang J. Yang D. S. Li M. X. Shi L. X. (2016). Metabolic profiles reveal changes in wild and cultivated soybean seedling leaves under salt stress. PloS One 11, e0159622. doi: 10.1371/journal.pone.0159622 27442489 PMC4956222

[B91] Zhang Y. Q. Yang F. Ren H. J. Liu J. L. Mu W. Y. Wang Y. (2019). *Pseudomonas monteilii* PN1: a great potential p-nitrophenol degrader with plant growth promoting traits under drought and saline-alkali stresses. Biotechnol. Lett. 41, 801–811. doi: 10.1007/s10529-019-02692-4 31089841

[B92] Zuluaga M. Y. A. Milani K. M. L. Miras-Moreno B. Lucini L. Valentinuzzi F. Mimmo T.. (2021). Inoculation with plant growth-promoting bacteria alters the rhizosphere functioning of tomato plants. Appl. Soil Ecol. 158, 103784. doi: 10.1016/j.apsoil.2020.103784

