# Supplementary material for: Sphingomonas sp. Hbc-6 alters physiological metabolism and recruits beneficial rhizosphere bacteria to improve plant growth and drought tolerance
Source: Front Plant Sci. 2022 Oct 28;13:1002772. doi: 10.3389/fpls.2022.1002772 (PMC9650444; doi:10.3389/fpls.2022.1002772)
Supplement: Supplementary file 1 [file DataSheet_1.docx]

Supplementary Table

**Table S1**. **Effects of different treatments on Maize seed germination.** GR represents germination rate, GE represents germination energy. Different letters indicate significant differences following the ANOVA Duncan test (*P* < 0.05).

| **Treatments** | **GR (%)** | **GE (%)** |
| --- | --- | --- |
| 0% PEG6000 control | 96.08 ± 3.40 a | 86.28 ± 3.40 c |
| 0% PEG6000 Hbc-6 | 100.00 ± 0.00 a | 97.78 ± 3.85 a |
| 5% PEG6000 control | 95.14 ± 1.20 a | 83.33 ± 4.17 c |
| 5% PEG6000 Hbc-6 | 97.78 ± 3.85 a | 97.78 ± 3.85 a |
| 10% PEG6000 control | 90.00 ± 3.46 b | 74.67 ± 4.62 d |
| 10% PEG6000 Hbc-6 | 100.00 ± 0.00 a | 97.78 ± 3.85 a |
| 15% PEG6000 control | 82.22 ± 1.93 c | 70.00 ± 3.33 d |
| 15% PEG6000 Hbc-6 | 91.11 ± 7.70 b | 86.67 ± 6.67 c |

**Table S2**. **Differential metabolites in Maize under normal condition (MC vs. WH).** Differential metabolites in maize plants identified by VIP selection method (VIP ≥ 1) and fold-change analysis (fold change ≥ 2 or fold change ≤ 0.5).

| **Class** | **Compound** | **VIP** | **Fold-change** | **modulation** |
| --- | --- | --- | --- | --- |
| Alkaloids | N-Feruloyl serotonin | 1.45E+00 | 1.91E-01 | down |
|  | Melatonin | 1.17E+00 | 2.04E-01 | down |
| Amino acid  and derivatives | 3-(6-Hydroxy-3,4-dioxo-1,5-cyclohexadien-1-yl)-L-alanine | 1.10E+00 | 4.91E-01 | down |
|  | L-(-)-Cystine | 1.24E+00 | 2.76E-01 | down |
|  | (-)-3-(3,4-Dihydroxyphenyl)-2-methylalanine | 1.50E+00 | 4.47E-06 | down |
|  | Glutathione | 1.87E+00 | 2.44E+00 | up |
|  | N-acetylglycine | 1.68E+00 | 4.84E-01 | down |
| Flavanone | Phloretin | 1.52E+00 | 7.37E+00 | up |
| Flavone | Eriodictiol 6-C-hexoside 8-C-hexoside-O-hexoside | 1.09E+00 | 3.63E-01 | down |
|  | Apigenin | 1.59E+00 | 3.10E-01 | down |
| Flavonoid | Quercetin | 1.96E+00 | 2.31E-01 | down |
|  | Morin | 1.94E+00 | 1.93E-01 | down |
|  | Glabridin | 1.12E+00 | 3.86E-01 | down |
| Flavonol | Kaempferol | 2.14E+00 | 1.22E-01 | down |
|  | Isorhamnetin | 1.51E+00 | 4.50E-01 | down |
|  | Rhamnetin (7-O-methxyl quercetin) | 1.15E+00 | 4.95E-01 | down |
| Isoflavone | Biochanin A | 1.07E+00 | 1.43E-01 | down |
| Lipids | 4-oxo-9Z,11Z,13E,15E-octadecatetraenoic acid | 1.70E+00 | 4.77E-01 | down |
|  | MAG (18:4) isomer3 | 1.28E+00 | 4.74E-01 | down |
|  | PC 19:2/16:0 | 1.48E+00 | 2.54E+00 | up |
|  | 12,13-EODE | 1.01E+00 | 3.77E-01 | down |
| Nucleotide  and derivates | Xanthine | 2.17E+00 | 4.01E-04 | down |
|  | Guanine | 1.06E+00 | 4.52E-01 | down |
| Organic acids  and derivatives | Sinapoyl malate | 1.32E+00 | 4.86E-01 | down |
|  | 2-Aminoethanesulfonic acid | 2.15E+00 | 6.38E-04 | down |
|  | Methyl gallate | 1.53E+00 | 3.78E-01 | down |
|  | lithospermic acid B | 2.18E+00 | 6.91E-04 | down |
| Phenolamides | "N',N""-p-coumaroyl-feruloyl putrescine" | 1.49E+00 | 1.03E-03 | down |
|  | 1,5-Diaminopentane | 1.16E+00 | 6.06E-02 | down |
| Phenylpropanoids | Homovanillic acid | 1.48E+00 | 3.94E-01 | down |
|  | Syringin | 1.64E+00 | 2.79E-01 | down |
|  | Resveratrol | 2.18E+00 | 5.36E+03 | up |
|  | 6-MethylCoumarin | 1.66E+00 | 2.26E+00 | up |
|  | Esculetin (6,7-dihydroxycoumarin) | 1.72E+00 | 3.79E-01 | down |
|  | Imperatorin | 1.27E+00 | 1.82E-01 | down |
| Polyphenol | Theaflavin | 1.94E+00 | 1.33E-01 | down |
| Quinones | Purpurin | 2.18E+00 | 3.08E-03 | down |
| Terpene | Phytocassane D | 1.97E+00 | 4.69E-01 | down |
|  | Oleanolic acid | 1.51E+00 | 4.20E-01 | down |
| Vitamins  and derivatives | Riboflavin | 1.30E+00 | 4.32E-01 | down |
| Others | α-Ionone | 1.84E+00 | 4.90E-01 | down |
|  | beta-Zearalanol | 2.16E+00 | 4.95E-04 | down |
|  | Pectin (Technical Grade) | 1.79E+00 | 2.39E+00 | up |

**Table S3**. **Differential metabolites in Maize under medium drought (DMC vs. MH).** Differential metabolites in maize plants identified by VIP selection method (VIP ≥ 1) and fold-change analysis (fold change ≥ 2 or fold change ≤ 0.5).

| **Class** | **Compound** | **VIP** | **Fold-change** | **modulation** |
| --- | --- | --- | --- | --- |
| Alkaloids | Cinnamoyl tyramine | 1.24E+00 | 3.59E-01 | down |
|  | Cocamidopropyl betaine | 1.37E+00 | 3.95E-01 | down |
|  | (-)-Cotinine | 1.41E+00 | 4.51E-01 | down |
| Amino acid  and derivatives | Glutathione | 1.78E+00 | 2.14E+00 | up |
|  | 1-Aminocyclopropanecarboxylic acid | 1.07E+00 | 2.12E+00 | up |
| Flavanone | Naringenin | 1.84E+00 | 4.79E-01 | down |
|  | Naringenin chalcone | 1.91E+00 | 4.95E-01 | down |
|  | Butein | 1.02E+00 | 2.47E+00 | up |
|  | Homoeriodictyol | 1.65E+00 | 3.84E-01 | down |
| Flavone | Tricin 5-O-acetylglucoside | 1.07E+00 | 2.93E+00 | up |
|  | C-hexosyl-chrysoeriol O-hexoside | 1.35E+00 | 4.01E-01 | down |
|  | Chrysoeriol O-hexosyl-O-pentoside | 1.31E+00 | 4.88E-01 | down |
|  | 8-C-hexosyl-luteolin O-pentoside | 1.48E+00 | 2.68E-01 | down |
|  | Chrysoeriol C-hexosyl-O-rhamnoside | 1.40E+00 | 3.29E-01 | down |
|  | Tricin 4'-O-(syringyl alcohol) ether 5-O-hexoside | 1.65E+00 | 2.42E+00 | up |
|  | Tricin 4'-O-syringic acid | 1.84E+00 | 2.03E+00 | up |
|  | Chrysoeriol 8-C-pentosyl-O-rutinoside | 1.23E+00 | 4.14E-01 | down |
|  | Apigenin O-hexosyl-O-rutinoside | 2.05E+00 | 1.12E-03 | down |
|  | Luteolin | 1.90E+00 | 4.12E-01 | down |
|  | Apigenin | 1.11E+00 | 3.35E-01 | down |
|  | Butin | 1.78E+00 | 4.62E-01 | down |
| Flavonoid | Vestitol | 1.79E+00 | 2.19E+01 | up |
|  | Quercetin | 1.88E+00 | 1.88E-01 | down |
|  | Morin | 1.90E+00 | 1.68E-01 | down |
| Flavonol | Kaempferol | 1.82E+00 | 1.47E-01 | down |
|  | Isorhamnetin | 1.92E+00 | 2.45E-01 | down |
|  | Dihydromyricetin | 2.07E+00 | 1.08E-04 | down |
| Isoflavone | 2'-Hydroxygenistein | 1.95E+00 | 4.92E-01 | down |
|  | Formononetin 7-O-glucoside (Ononin) | 1.28E+00 | 2.18E+00 | up |
| Organic acids  and derivatives | Maleic acid | 1.19E+00 | 2.18E+00 | up |
|  | Benzoylformic acid | 1.52E+00 | 4.60E-01 | down |
|  | Citraconic acid | 1.86E+00 | 3.42E+00 | up |
|  | lithospermic acid B | 2.06E+00 | 5.63E-04 | down |
|  | Phosphoenolpyruvate trisodium salt | 1.25E+00 | 2.73E+00 | up |
| Phenolamides | N-Acetyl tryptamine | 1.33E+00 | 2.34E-01 | down |
|  | Putrescine | 1.98E+00 | 2.08E+00 | up |
| Phenylpropanoids | O-Feruloyl 3-hydroxylcoumarin | 1.48E+00 | 2.06E+00 | up |
|  | O-Caffeoyl maltotriose | 2.07E+00 | 8.72E-04 | down |
|  | Caftaric acid | 1.30E+00 | 4.86E-01 | down |
|  | Resveratrol | 1.10E+00 | 3.55E+00 | up |
|  | 6-Hydroxy-4-methylcoumarin | 1.38E+00 | 2.57E+00 | up |
|  | Zingerone | 1.93E+00 | 4.90E-01 | down |
|  | Phillyroside | 2.07E+00 | 4.95E-04 | down |
| Others | Polydatin | 1.01E+00 | 2.40E+00 | up |
|  | beta-Zearalanol | 2.06E+00 | 7.47E-04 | down |
