# Supplementary material for: Sphingomonas sp. Hbc-6 alters physiological metabolism and recruits beneficial rhizosphere bacteria to improve plant growth and drought tolerance
Source: Front Plant Sci. 2022 Oct 28;13:1002772. doi: 10.3389/fpls.2022.1002772 (PMC9650444; doi:10.3389/fpls.2022.1002772)
Supplement: Supplementary file 2 [file Presentation_1.pdf]

Supplementary Material

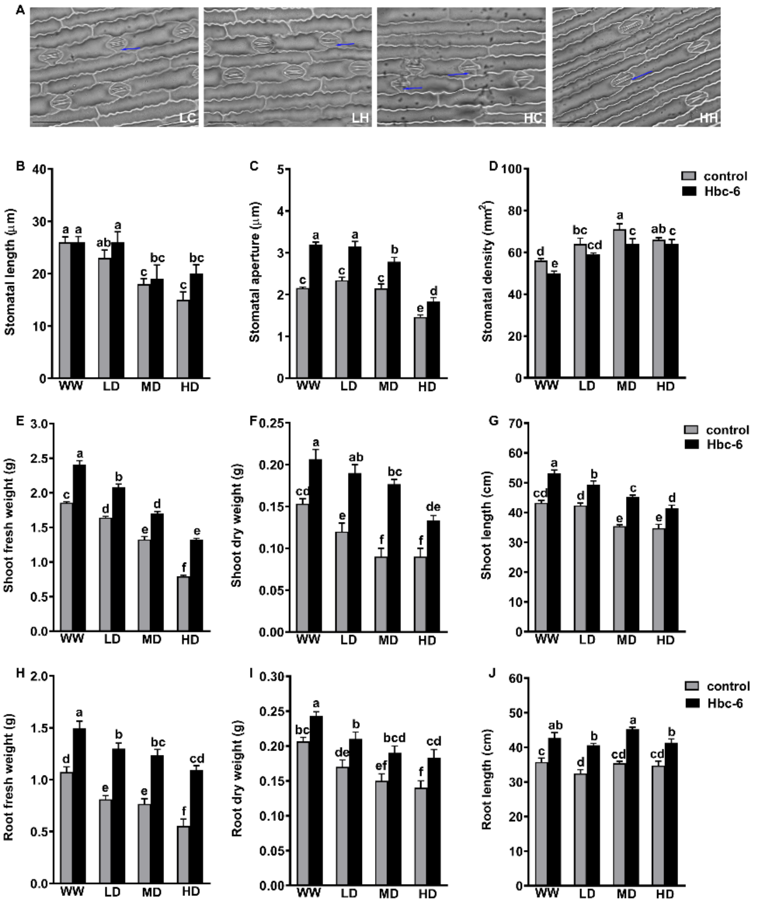

Supplementary Figure 1. Effects of *Sphingomonas* sp. Hbc-6 and different drought gradients on stomatal structure, above- and below-ground mass of maize after 26 days of continuous inoculation Hbc-6. (A) Representative images of stoma on different drought degree treatments, the scale bars represent 100 μm. The blue arrow represent the subsidiary cell of maize. (B) Stomatal length, (C) stomatal aperture, (D) stomatal density, (E) shoot fresh weight, (F) shoot dry weight, (G) shoot length, (H) root fresh weight, (I) root dry weight and (J) root length of inoculated with Hbc-6 and non-inoculated (control) plant under drought gradients were measured after 25 days. Four different drought degree treatment were normal condition (WW), light drought (LD), medium drought (MD) and serious drought (HD). LC represents non-inoculated (control) plant under light drought, LH represents inoculating with Hbc-6 under light drought, HC represents non-inoculated (control) plant under serious drought and HH represents inoculating with Hbc-6 under serious drought. Data are mean ± SD of three independent experiments (leaves from three plants). Different letters indicate statistically significant difference compared with control-treated roots (ANOVA Duncan test; $P < 0.05$).

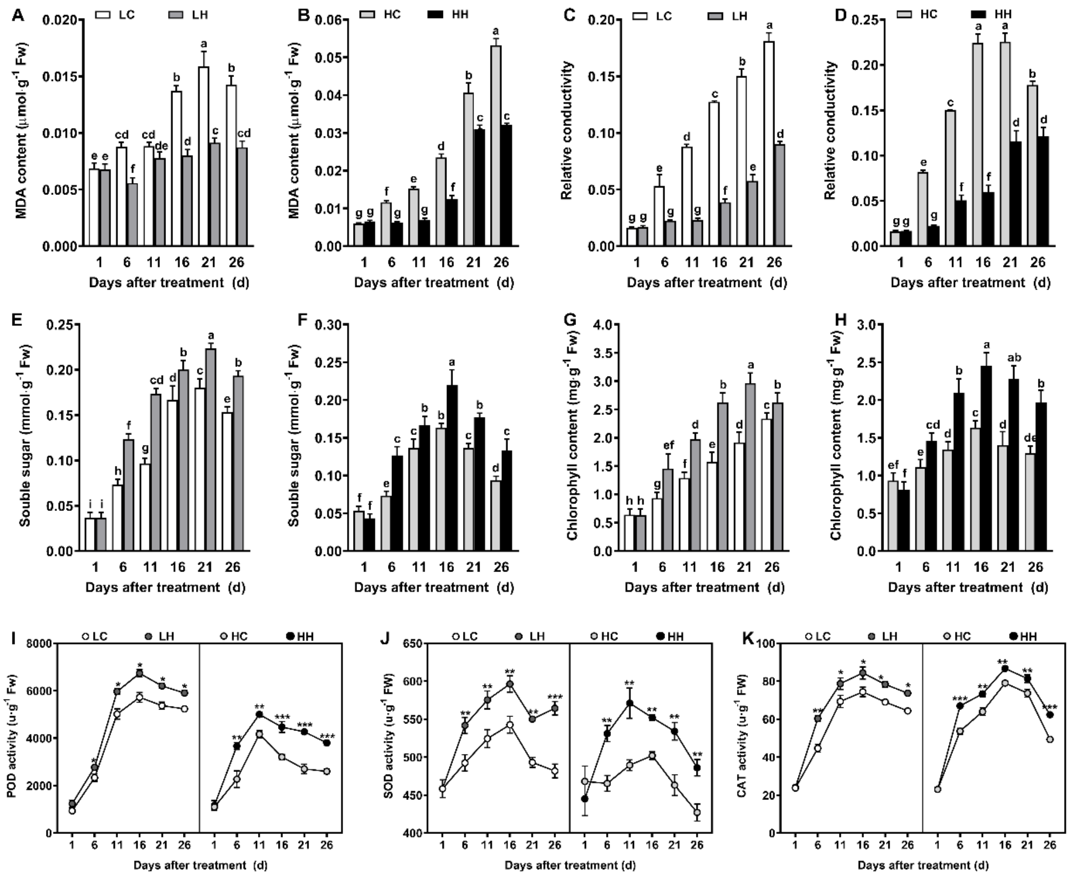

Supplementary Figure 2. Physiological and antioxidant system responses of maize to *Sphingomonas* sp. Hbc-6. Changes of (A-B) MDA content, (C-D) relative conductivity, (E-F) soluble sugar and (G-H) chlorophyll content in maize were measured with treatment time under different treatments. Time course of (I) POD, (J) SOD and (K) CAT in response to Hbc-6. Different letters/asterisks indicate statistically significant differences at ANOVA Duncan test; $P < 0.05$; * represent $P < 0.05$; ** represent $P < 0.01$; *** represent $P < 0.001$).

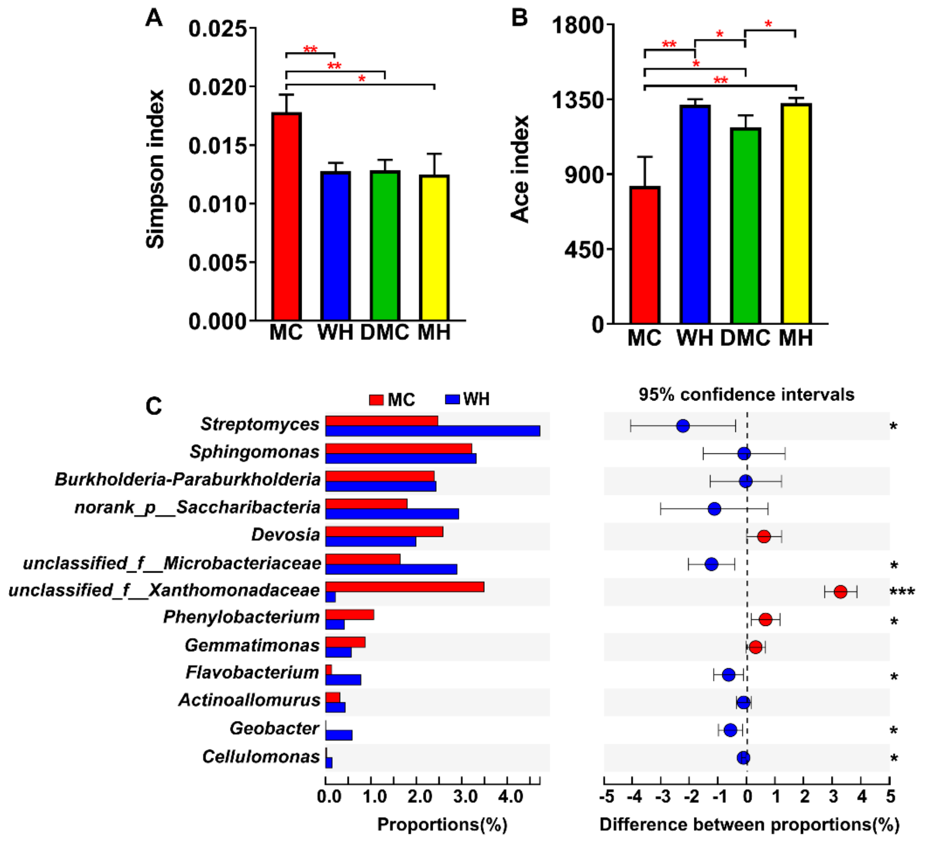

Supplementary Figure 3. Effects of *Sphingomonas* sp. Hbc-6 on rhizosphere bacterial community diversity and composition. (A) Simpson and (B) Ace index of inoculated with Hbc-6 and non-inoculated (control) plant under normal condition and medium drought. (C) Differential abundance of species inoculated with Hbc-6 and non-inoculated (control) rhizosphere soil under normal condition at the genus level. The x-axis represents different groups, boxes of different colors represent different groups, and the y-axis represents the average relative abundance of a species in different groups. Different asterisks indicated significant differences following the student's t-test (* represent P < 0.05; ** represent P < 0.01; *** represent P < 0.001).
